# Molecular docking analysis of doronine derivatives with human COX-2

**DOI:** 10.6026/97320630016483

**Published:** 2020-06-30

**Authors:** Sardar Hussain, Chandrasekharan Guruvayoorappan, KP Komal, Sreenivasa Ennaganti

**Affiliations:** 1 Department of Biotechnology, Government Science College, Chitradurga - 577501, Karnataka; R and D Centre, Bharathiar University, Coimbatore 641046, Tamilnadu, India; 2 Laboratory of Immuno pharmacology and Experimental Therapeutics, Division of Cancer Research, Regional Cancer Centre, Medical College Post, Thiruvananthapuram - 695011, Kerala, India; 3 Department of Biochemistry, Government Science College, Chitradurga 577501, Karnataka ,India; 4 Research Associate, Department of Bioinformatics, Averin Biotech, Hyderabad, 500044, Telangana,India

**Keywords:** Emilia sonchifolia, doronine, inflammation, phyto-compounds, pyrrolizidine alkaloid

## Abstract

Cyclooxygenase-2 (COX-2) is linked to inflammation. Therefore, it is of interest to design and develop novel inhibitors for COX-2. Hence, we report the molecular docking based binding features of doronine derivatives (desacetyldoronine, floradnin, onetine, 22310115, 21159807) with the human Cyclooxygenase-2 as potential inhibitors. A pyrrolizidine alkaloid doronine a molecular constituents of Emilia sonchifolia is an herbal alternative to known drugs in the prophylaxis of inflammation. We report the molecular docking, pharmacophore, ADMET and molecular properties analysis data of doronine and its similar compounds. Docking and ADMET Data shows that COX-2 binds with doronine with optimal features for further consideration.

## Background

Inflammation is linked with the excretion of chemicals substances called as mediators such as histamines,bradykinin,5-hydroxytryptamine,interleukin-1(IL-1),prostaglandins,leukotrienes (LTs), enzymesetc, [1,2].

These substances develop certain cellular effects that vitally participate in persistence, genesis, and the pain severity accompanying infection, or trauma [3]. Inflammation should be temporary, however, under certain conditions the acute response leads to being chronic along with diseases like diabetes, cancer, cardiac, Alzheimer's and other neurological disorders [4]. Medicines used are known to be of non-steroidal or steroidal therapeutics. NSAIDs do have the anti-inflammatory impact by constraining the COX enzyme [5]. A decrease in the pro inflammatory cytokines triggered by glucocorticoids along with rise in anti-inflammatory cytokines ensuing higher activity of anti-inflammation is known [6]. One of the critical issues with these drugs is that they retain various detrimental after-effects and build resistance in case of chronic use [7,8]. The best alternative to these drugs is the naturally occurring products that aid in the recognition of lead components that could substitute the chemically available therapeutics for inflammatory diseases [9,10].

A large resource of raw materials to screen and develop new components having pharmacological activity, without adverse effects at low cost is available [11, 12]. Herbal drugs, phyto-medicines act as a precursor for synthetic analogues [13]. Alkaloids are documented for its laxative, anti-tumor, anti-cholinergic, diuretic, antiviral, sympatho-mimetic, antihypertensive, anti-depressant, hypno-analgesic, mio-relaxant, antimicrobial, anti-tussigen, and anti-inflammatory activities [14,15,16]. Alkaloids with pyrrolizidine nucleus make an interesting set of molecules is relevant for human and animal nutrition along with pharmacological and toxicological features [17].

Pyrrolizidine alkaloids (PA) are few of the naturally existing heterocyclic organic compounds, found in 6,000 species of plants representing 3% of world flora in the form of secondary metabolites [17,18]. They are derived mostly from ornithine and around 95% are found in few plant taxas like Eupatorieae tribes, Senecioneae tribes, genera of Boraginaceae, Crotalaria (Fabaceae), and Orchidaceae family. They can be found in plants either as a free form of pyrrolizidine and as pyrrolizidine alkaloids N-oxides (PANOs) [19]. These are ester compounds derived through 5-membered ring (necine) in form of di and mono-cyclic diesters. PAs with necine having a double bond at 1,2 and a non-substitution near to the N2 atom is harmful for animals and humans [20]. PAs with double bond at the necine base is linked to higher toxicity compared to compounds with saturated necine base [19].

Many plants possessing pyrrolizidine alkaloids with anti-inflammatory nature were screened and analyzed like in Emilia sonchifolia, an annual herbaceous plant [21]. It has medicinal benefits in treating diarrhoea, night blindness, sore throat, rashes, measles, inflammatory diseases, eye and ear ailments, fever, stomach tumor, malaria, asthma, liver diseases, eye inflammation, earache, and chest pain. The aerial part is believed to contain flavonoids, terpenes and alkaloids [22,23]. Pyrrolizidine alkaloids, senecionine, seneciphylline, integerrimine, senkirkine, otosenine, neosenkirkine, petasitenine, acetylsenkirkine, acetyl petasitenine, desacetyldoronine, and doronine have been identified from E. sonchifolia. Nonetheless, due to enormous distribution of these plants, the components of PAs are contained as a public health issue due to their adverse effects along with hepatotoxicity [24,25,26]. Therefore, it is of interest to design and develop novel inhibitors for COX-2. Hence, we report the molecular docking based binding features of doronine derivatives (desacetyldoronine, floradnin, onetine, 22310115, 21159807) with the human Cyclooxygenase-2 as potential inhibitors.

## Materials and Methods:

### COX-2 structure:

**Figure 1:**
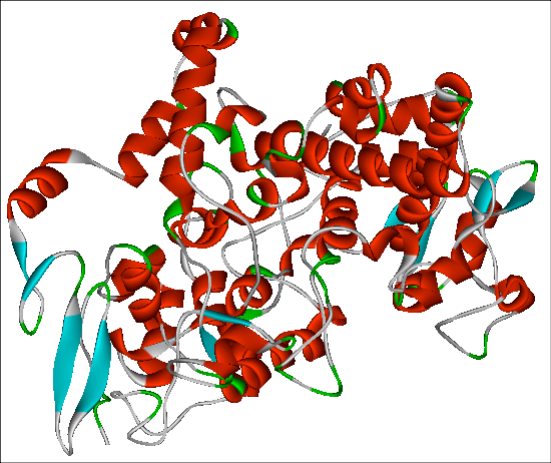
The structure of the human COX-2.

The structure of human COX-2 (Figure 1) in complex with an aspirin was downloaded from the PDB with PDB ID: 5F19 at 2.04 Å resolution and R-value of 0.168. The structure was adequately processed using the CHARMm force field for further studies.

### Identification of active site:

Active sites in COX-2 were identified using the DS Analyze Binding Site tool with default parameters.

### Ligand preparation:

**Figure 2:**
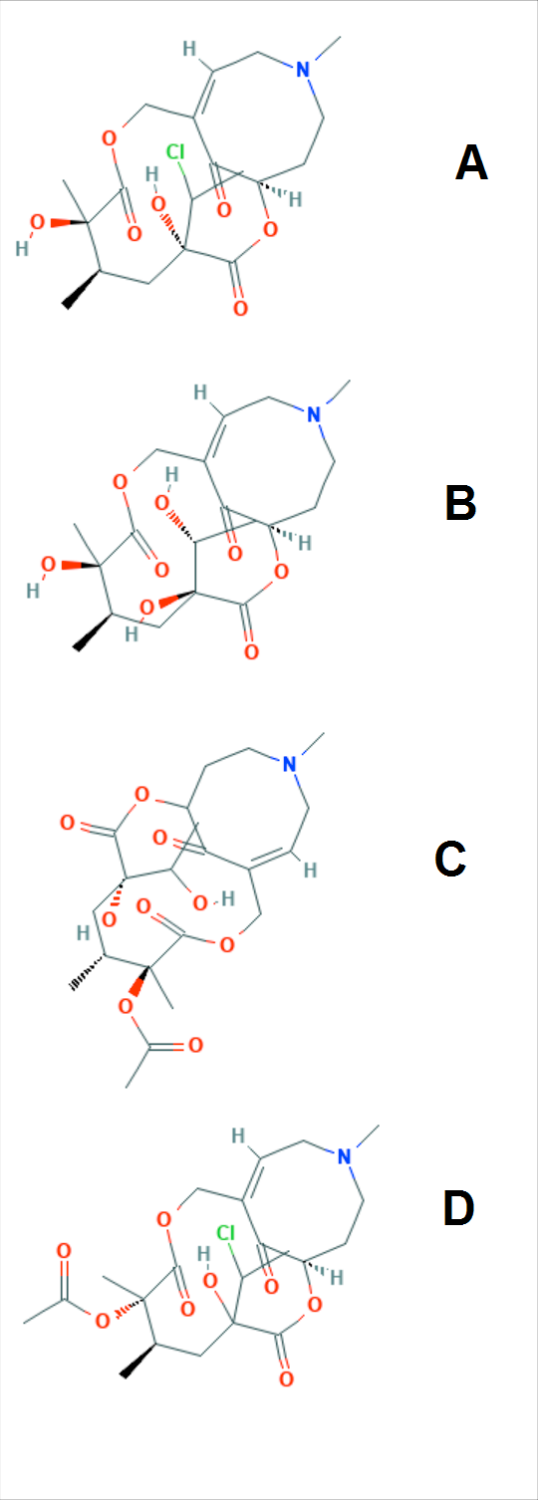
The structure of the PA compounds.

The two dimensional structures of PA compounds (Figure 2) are drawn with the aid of ACD/ ChemSketch (12.0) and are later imported in to ADS. Ligand compounds were processed with the CHARMm force field using the DS protocol 'prepare ligands' following standard procedures.

### Docking studies:

Molecular docking based binding features of doronine derivatives (desacetyldoronine, floradnin, onetine, 22310115, 21159807) with the human Cyclooxygenase-2 as potential inhibitors was gleaned using LibDock in the Discovery Studio Software.

### Pharmacophore model generation and validation:

Two different methods are applied for the pharmacophore model generation using DSt: (1) Ligand (common feature approach) and (2) structure based pharmacophore modeling, to analyse the fitting of the designed compounds to the generated pharmacophores.

### Ligand based pharmacophore modeling:

Common feature for pharmacophore modeling is applied with PA compounds using the HipHop algorithm in DS. A maximum of 255 conformations are created per compound using the FAST conformer method within an energy range of 20 kcal/mol over the global energy minimum.

### Structure based pharmacophore modeling:

Ludi interaction maps were generated to study ligand-target interaction.

### ADMET prediction:

Adsorption, distribution, metabolism, excretion and toxicity (ADMET) descriptors were collected using the DS tool.

### Molecular properties analysis:

Molecular properties such as molecular weight, number of hydrogen bonds that would be donated or accepted, an octanol water partition coefficient (log P), number of rotatable bonds, number of rings, number of aromatic rings and molecular functional polar surface area of all the compounds are calculated using the DS tool.

## Results and discussion:

Molecular docking studies of the PA compounds comapred with the standard drug celecoxib were completed using the default parameter of LibDock to explore the binding pattern with the human COX-2 (PDB ID: 5F19). Docking results are analyzed using the docking scores, binding modes and interaction of each compound with the functional residues of COX-2 protein. LibDock produces several poses, each producing their corresponding LibDock scores with different orientations within the defined active site of the COX-2 protein. The high LibDock score of the ligand pose was taken into account for the prediction of the best ligand binding conformation. The ligands with high LibDock scores are preferred for estimating binding energies of the protein–ligand complex. Binding poses with highest LibDock Score and lowest binding energy are preferred as the best pose and further binding interactions of the best pose for each compound are analysed. So, the above pre-validated analysis was used to sort out the retrieved hit molecules and then those are further validated by using the visualization method to find the suitable binding mode of the ligand based on the critical interactions with the active site residues. The docked compounds were found to have similar binding poses to the co-crystallized ligand, thus validating the adopted docking methodology. Finally, the Analyze Ligand Poses subprotocol was performed to count H bonds and close contacts (van der Waals clashes) between the poses and human COX-2. Table 1 depicts the LibDock scores, interaction data and binding energies for PA compounds.

**Figure 3:**
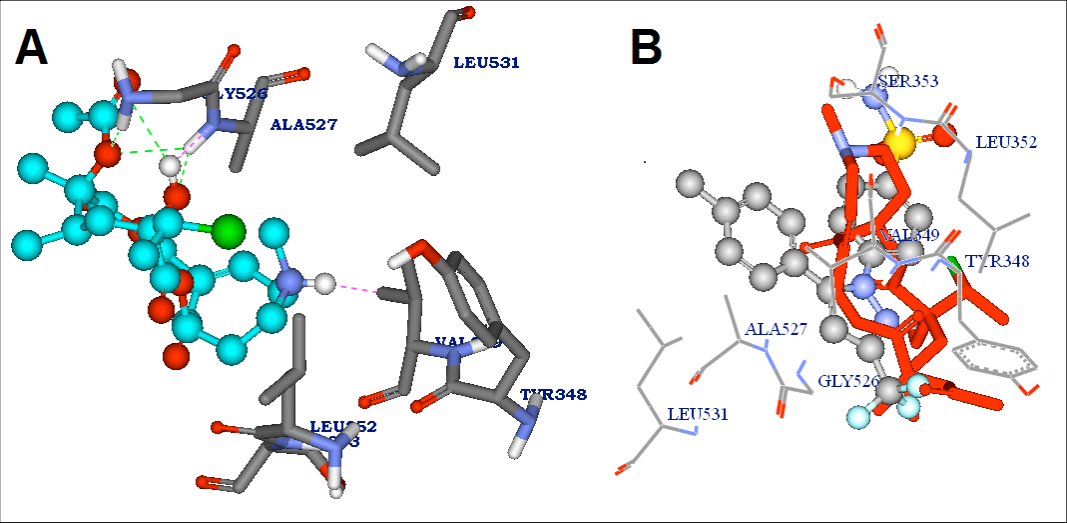
Shows Receptor-ligand interactions of (a) Doronine (b) celecoxib with active site residues of human COX-2

It is observed that all the docked compounds exhibited fitness scores with a range of 120.153 to 95.784 and that for the drug celecoxib is found with a score of 141.165. Among all the compounds, compound doronine was ranked highest with a docking score of 120.153 and binding energy of -3.65569  kcal/mol which showed a good agreement with the celeoxib docking score 141.165 and binding energy -5.29076. Further, the compound doronine was studied in detail in order to extract useful information about the compound conformations in the active pocket of the human COX-2 enzyme. It also showed good interactions with the binding site residues of target protein in similar pattern of celecoxib. The protein–ligand interaction visualization of the compound doronine and the drug celecoxib is shown in the Figure 3. This compound was docked into the active site region making three hydrogen interactions. First hydrogen bond is formed with the 31st hydrogen atom of the compound interacted with the nitrogen atom of Glycine 526 (Doronine:H32 - B:GLY526:N) with a hydrogen bond distance of 2.404000 Å Second hydrogen bond is formed with the first hydrogen atom of amine group of Glycine 526 interacted with the oxygen atom of the compound (B:GLY526:HN1 - Doronine:O9) with a hydrogen bond distance of 2.483 Å. The third hydrogen bond is formed between the hydrogen atom of amine group of Alanine 527 and the second oxygen atom of the compound (B: ALA527:HN - Doronine:O2) with a hydrogen bond distance of 2.464 Å. It was observed that some close interactions are formed with the amino acid residues VAL349.

**Figure 4:**
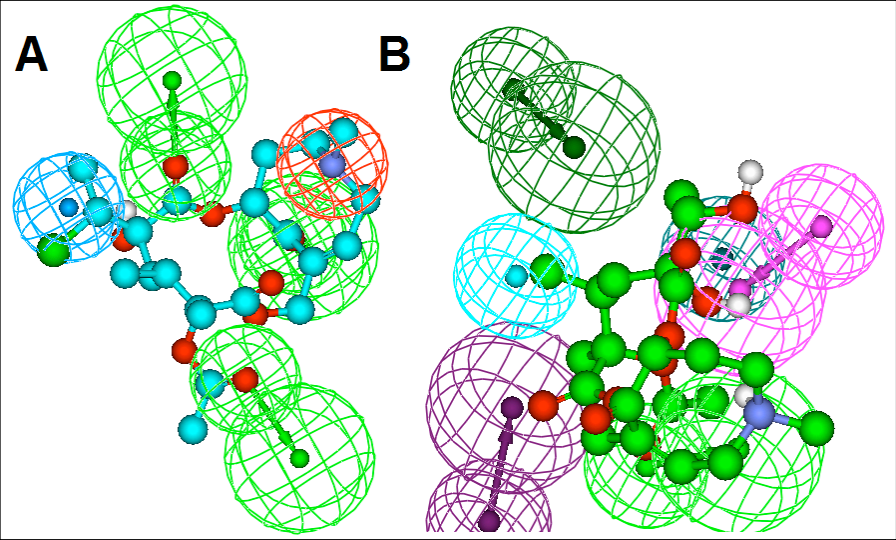
Mapping of (a) Doronine with Hypo 1; (b) Floradnin with protein human COX-2 model.

The HipHop algorithm computes ten common pharmacophore hypotheses and Hypo1 is considered as the most reliable pharmacophore hypothesis containing three HBD and one Hydrophobe and one positive ionizable feature. All the compounds are mapped on to the Hypo1, ranked according to their fit values ([Table T2]2) and the compound doronine fitted well on the pharmacophore with a high fitvalue of 5 (Figure 4a). The Interaction Generation protocol constructed a structured based pharmacophore model of our protein protein human COX-2 based on the active site residues inside the sphere. The final edited pharmacophore model has two HBD, two HBA and two hydrophobic features. Using ligand pharmacophore mapping, compounds are mapped and ranked according to the fit values (Table 3). Based on the fit values, the compound Floradnin fitted well on the pharmacophore with a fitvalue of 1.812 (Figure 4b).

**Figure 5:**
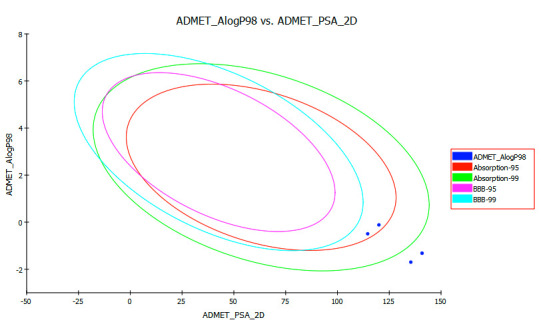
ADMET biplot curve showing the 95% and 99% confidence limit ellipse corresponding to the blood-brain barrier and intestinal absorption model.

ADMET properties are an important index to check whether clinical candidates can reach the required standard. ADMET studies of the compounds predicted using ADMET descriptor module of DS to provide insight into the pharmacokinetic property of the compounds. Table 4 shows the ADMET results of the compounds. According to the Discovery Studio parameters, standard analysis of an ideal drug like compound is as follows: level 0 for human intestinal absorption, level 3 and level 4 for solubility, level 0 for non-inhibitory property with CYP450 2D6, level 3 for BBB penetration and level 0 for non-toxicity. ADMET descriptors, the 2D polar surface area in A2 per compound are plotted against their consonant estimated atom-type partition coefficient (ALogP98). The biplot curve consisted of two ellipses containing of 95% and 99% confidence levels for blood-brain barrier penetration and human intestinal absorption models. These ellipses elucidate zones where well-occupied compounds are settled. The compounds are found to be in the range of 95 and 99 % confidence ellipse for both the intestinal absorption and BBB shown in Figure 5. The polar surface area (PSA) has an important role for human intestinal absorption and membrane permeability. The curve showed that PSA has an inverse relationship with intestinal absorption and membrane crossing. Due to higher PSA of all the compounds they have high tendency towards more intestinal absorption and very low blood-brain barrier penetration and so have high oral bioavailability. Predicting the value of AlogP98 can determine the hydrophilicity of the compound. AlogP98 < 5 may be related to the absorption or permeability of the compound. Considering the AlogP98 criteria, all PAs had AlogP98 value <5, that has also in turn accepted the 99% and 95% confidence ellipse for both intestinal absorption and blood-brain barrier penetration.

All the compounds shown a BBB level of 4 showing undefined penetration across the Central Nervous System (CNS) hence it lessen the side effects linked to CNS. The found absortion level was 0 for the compounds Doronine and Desacetyldoronine revealing good intestinal absorption whereas level 2 for the compounds 22310115, Floradnin and Onetine indicating low absorption. For all the compounds, the calculated hepatotoxic level was 1 implying the compounds as toxic. All the compounds are found to be having the solubility level 4 except doronine found to have 3. The solubility level 3 indicating very good solubility, level 4 indicating the best or most favourable solubility. Similarly, compounds having level 0 was found to be satisfactory with respect to CYP 450 2D6 liver enzyme, suggesting that the compounds are non-inhibitor of the metabolic enzyme and Finally, the PPB value found to be 0 for all of the compounds which denotes the compound have binding ≤90 % clearly revealing that the compound have good bioavailability and are not likely to be highly bound to carrier proteins in the blood.

Molecular physicochemical and drug likeness are the two attributes, which provide base for the compound to be a efficient drug candidate. The compounds were evaluated, followed the Lipinski's rule of five. Pharmaceutical chemists in drug design and development to predict oral bioavailability of potential lead or drug molecules commonly use Lipinski's rule of five. Concerning the standard rule of five, a candidate molecule will likely to be orally active, if the calculated octanol/water partition coefficient (Log P) value less than 5, favorable range of molecular weight is between 160-480 g/mol, number of rotatable bonds is 15 or less than 15, number of hydrogen bond acceptors (nitrogen or oxygen atoms) be 10 or less than 10 and hydogen bond donors (nitrogen or oxygen atoms with one or more hydrogen atoms) values should 5or less than 5, is preferable for drug likeness properties.. The molecular properties of the compounds are calculated by using DS are presented in Table 5. According to this, compounds in this study have well qualified with all the rules of Lipinski's filter. Log P (an octanol water partition coefficient) is applied as significant tool in quantitative structure activity relationship (QSAR) studies and also in rational drug design as a measure of molecular hydrophobicity or lipophilicity. Log P values of all the compounds were found to be less than 5 and are in clear acceptance of Lipinski's rule of five, suggesting permeability across cell membrane justifying their oral use. Molecular weight of all the compounds was found to be less than 500 and thus these molecules are easily transported, diffused and absorbed as compared to large molecules. Number of hydrogen bond acceptors (O and N atoms) and number of hydrogen bond donors (NH and OH) in the compounds were in accordance with the Lipinski's rule of five i.e. less than 10 and 5 respectively. It can be predicted that the compounds are likely to be orally active as they obeyed Lipinski's rule of five.

## Conclusions:

We report the molecular docking based binding features with ADMET data of doronine derivatives (desacetyldoronine, floradnin, onetine, 22310115, 21159807) with the human Cyclooxygenase-2 for further consideration in the context of inflammation.

## Figures and Tables

**Table 1:** Calculated docking scores, binding energies and hydrogen bond interactions along with their bond lengths of the targeted PA compounds inside human COX-2 active site.

Name	Libdock score	Interacting	Binding Energy	H bond	Distance
		Amino acids			
Doronine	120.153	Tyr348,Val349	-3.65569	Doronine:H32 - B:GLY526:N	2.404
		Leu352,Ser353		B:GLY526:HN1 - Doronine:O9	2.483
		Leu531,Gly526		B:ALA527:HN - Doronine:O2	2.464
		Ala527		Doronine:H33 - B:VAL349:CG1	1.738
				Doronine:H32 - B:ALA527:N	2.084
				Doronine:H32 - B:ALA527:HN	1.368
22310115	98.14	Tyr348,Val349	161.81019	B:LEU531:HN2 - 22310115:O9	1.993
		Leu352,Ser353		22310115:H33 - B:LEU531:N	1.661
		Leu531,Gly526		B:LEU531:HN2 - 22310115:H33	1.613
		Ala527			
Desacetyl	105.112	Tyr348,Val349	19.47484	B:GLY526:HN1 - Desacetyldoronine:O8	2.414
doronine		Leu352,Ser353		B:ALA527:HN - Desacetyldoronine:O8	2.238
		Leu531,Gly526			
		Ala527			
Floradnin	101.589	Tyr348,Val349	-1.47981	B:GLY526:HN1 - Floradnin:O5	1.736
		Leu352,Ser353		B:ALA527:HN - Floradnin:O2	2.471
		Leu531,Gly526		B:GLY526:HN1 - Floradnin:O5	1.736
		Ala527			
Onetine	95.784	Tyr348,Val349	23.92218	B:ALA527:HN - Onetine:O6	2.031
		Leu352,Ser353		B:GLY526:HN1 - Onetine:O5	2.308
		Leu531,Gly526			
		Ala527			

**Table 2 T2:** Predicted fit values of compounds from the common feature based hypothesis Hypo 1

Name	HBA_3	HBA_4	HBA_5	HYDROPHOBE	PosIonizable_1	Pharmprint	FitValue
Doronine	111	1	1	11111'	5
Doronine	111	0	1	'10111'	2.973
Desacetyldoronine	111	1	1	'11111'	4.326
Desacetyldoronine	111	0	1	'10111'	3.153
Onetine	111	1	1	'11111'	3.09
Onetine	111	0	1	'10111'	1.682
22310115	111	0	1	'10111'	2.658
22310115	111	1	1	'11111'	0.949
Floradnin	111	1	1	'11111'	2.158
Floradnin	111	1	1	'11111'	0.697

**Table 3:** The predicted fit values of compounds from the structure based pharmacophore model of human COX-2.

Name	HBA16	HBA59	HBD31	HBD74	Hydrophobe19	Hydrophobe51	FitValue	Pharmprint
Floradnin	0	1	0	1	1	0	1.812	'010110'
Floradnin	0	1	0	1	1	0	1.386	'010110'
Desacetyldoronine	0	1	1	0	1	0	1.806	'011010'
Desacetyldoronine	0	1	1	0	1	0	1.418	'011010'
22310115	0	1	1	0	1	0	1.424	'011010'
22310115	0	1	1	0	1	0	1.228	'011010'
Onetine	0	1	1	0	1	0	1.411	'011010'
Onetine	0	1	0	1	1	0	1.406	'010110'
Doronine	0	1	0	1	1	0	0.259	'010110'

**Table 4:** ADMET descriptors of the compounds

Name	BBB Level	Absorption Level	Solubility Level	Hepatotoxicity	CYP2D6	PPB Level	AlogP98	PSA 2D
Doronine	4	0	3	1	0	0	-0.116	120.013
22310115	4	2	4	1	0	0	-1.321	140.828
Desacetyldoronine	4	0	4	1	0	0	-0.495	114.597
Floradnin	4	2	4	1	0	0	-1.321	140.828
Onetine	4	2	4	1	0	0	-1.7	135.413

**Table 5:** Molecular properties of the PA compounds.

Name	ALogP	Molecular Weight	No of Acceptors	No of Donors	No of Rotatable Bonds
21159807	1.445	459.918	9	1	3
22310115	0.24	441.472	10	2	3
Desacetyldoronine	1.065	417.881	8	2	1
Doronine	1.445	459.918	9	1	3
Floradnin	0.24	441.472	10	2	3
Onetine	-0.139	399.435	9	3	1
